# Retraction Note: Molecular detection and quantification of *Plasmodium falciparum* gametocytes carriage in used RDTs in malaria elimination settings in northern Senegal

**DOI:** 10.1186/s12936-023-04636-w

**Published:** 2023-07-05

**Authors:** Kiswendsida Thierry Guiguemde, Yakou Dieye, Aminata Collé Lô, Magatte Ndiaye, Aminata Lam, Isaac Akhénaton Manga, Gnagna Dieng Sow, Moussa Diop, Tamba Souané, Marie Pièrre Diouf, Roger Clément Kouly Tine, Babacar Faye

**Affiliations:** 1 Department of Medical Parasitology, Medical Faculty, Cheikh Anta Diop University, Dakar, Senegal; 2 PATH, Malaria Control and Evaluation Partnership (MACEPA), Dakar, Senegal

**Retraction: Malaria Journal (2020) 19:123** https://doi.org/10.1186/s12936-020-03204-w

The Editor-in-Chief has retracted this article due to a flawed methodology. The study reported that gametocyte carriage could be detected by amplificating Plasmodium DNA retrieved from used RDTs. However, the method of amplifying DNA rather than RNA used meant that the authors detected *P. falciparum* parasite instead of the gametocytes. The Editor-in-Chief has therefore lost confidence in the validity of the results presented in this article. Furthermore, the authors were unable to provide sufficient documentation confirming that this study had received ethics approval.

Magatte Ndiaye agrees to this retraction. Kiswendsida Thierry Guiguemde, Yakou Dieye, Aminata Collé Lô, Isaac Akhénaton Manga, Gnagna Dieng Sow, Moussa Diop, Tamba Souané, and Babacar Faye have not responded to any correspondence from the editor/about this retraction. The editor was unable to obtain current email addresses for Aminata Lam, Marie Pièrre Diouf and Roger Clément Kouly Tine.

